# Physician workload associated with do-not-resuscitate decision-making in intensive care units: an observational study using Cox proportional hazards analysis

**DOI:** 10.1186/s12910-019-0355-0

**Published:** 2019-03-01

**Authors:** Kuan-Han Lin, Shu-Chien Huang, Chih-Hsien Wang, Chau-Chung, Tzong-Shinn Chu, Yen-Yuan Chen

**Affiliations:** 1 Graduate Institute of Medical Education & Bioethics, National Taiwan University College of Medicine, #1, Rd. Ren-Ai sec. 1, Taipei, 10051 Taiwan; 2 Department of Surgery, National Taiwan University Hospital, #7, Rd. Chong-Shang S, Taipei, 10002 Taiwan; 3 Department of Internal Medicine, National Taiwan University Hospital, #7, Rd. Chong-Shang S, Taipei, 10002 Taiwan; 4 Department of Medical Education, National Taiwan University Hospital, #7, Rd. Chong-Shang S, Taipei, 10002 Taiwan

**Keywords:** Do-not-resuscitate, Workload, Life-supporting treatment, Intensive care

## Abstract

**Background:**

Physicians play a substantial role in facilitating communication regarding life-supporting treatment decision-making including do-not-resuscitate (DNR) in the intensive care units (ICU). Physician-related factors including gender, personal preferences to life-supporting treatment, and specialty have been found to affect the timing and selection of life-supporting treatment decision-making. This study aimed to examine the influence of physician workload on signing a DNR order in the ICUs.

**Methods:**

This is retrospective observational study. The medical records of patients, admitted to the surgical ICUs for the first time between June 1, 2011 and December 31, 2013, were reviewed. We used a multivariate Cox proportional hazards model to examine the influence of the physician’s workload on his/her writing a DNR order by adjusting for multiple factors. We then used Kaplan–Meier survival curves with log-rank test to compare the time from ICU admission to DNR orders written for patients for two groups of physicians based on the average number of patients each physician cared for per day during data collection period.

**Results:**

The hazard of writing a DNR order by the attending physicians who cared for more than one patient per day significantly decreased by 41% as compared to the hazard of writing a DNR order by those caring for fewer than one patient (hazard ratio = 0.59, 95% CI 0.39—0.89, *P* = .01). In addition, the factors associated with writing a DNR order as determined by the Cox model were non-operative, cardiac failure/insufficiency diagnosis (hazard ratio = 1.71, 95% CI 1.00—2.91, *P* = .05) and the Therapeutic Intervention Scoring System score (hazard ratio = 1.02, 95% CI 1.00—1.03, *P* = .03). Physicians who cared for more than one patient per day were less likely to write a DNR order for their patients than those who cared for in average fewer than one patient per day (log-rank chi-square = 5.72, *P* = .02).

**Conclusions:**

Our findings highlight the need to take multidisciplinary actions for physicians with heavy workloads. Changes in the work environmental factors along with stress management programs to improve physicians’ psychological well-being as well as the quality.

## Background

In 1991, the Patient Self-Determination Act (PSDA) was passed in the United States to ensure that healthcare institutions informed patients of their rights to participate in their own medical decision-making and to complete advance directives [1]. Sensitive to the effect of the PSDA in the United States and the progress of hospice and palliative care, Taiwan became the first country in Asia to issue the “Hospice and Palliative Care Act” (HPCA) in 2000 [2]. This law gave patients with terminal illness, or whose death is inevitable in a short time as determined by attending physicians, the right to refuse unnecessary life-supporting treatment (LST) [3]. Furthermore, HPCA provided physicians a legal framework within which to sign do-not-resuscitate (DNR) orders in accordance with the will of patients.

In the past two decades, there has been plenty of studies on DNR orders and end-of-life care (EOLC) issues [4–8]. Many studies reported that increasing age, female gender, white race, single marital status, religious background, and the severity of clinical illness of patients are associated with writing a DNR order after admission to intensive care units (ICUs) [7–9]. In addition to patient-related factors, physician-related factors including gender, religious background, personal preferences to LSTs, and specialty also have been found to affect the timing and selection of LST decision-making [10–13]. Yuen et al. also reported that physicians’ failure to provide adequate information also prevented patients or surrogates from making DNR decisions [14]. Accordingly, physicians play a substantial role in facilitating communication regarding LST decision-making including DNR.

For Americans during their last year of life, approximately one quarter to one half are admitted to an ICU [15, 16], and about one in five deaths occurs in the ICU [17]. ICU is not only a place for critical care and LST decision-making, but it is also a highly stressful working environment for physicians. Because of caring for critically ill patients in the ICU, physicians have to deal with more end-of-life decision-making and communicate with family members of the patients. Physicians working in the ICU are found to have higher levels of stress due to work demands [18]. A multicenter study focusing on physicians working in ICUs showed that discrepancy for job demand, conflict, the ethical decision-making of withdrawing LSTs were all potential stressors from the work environment [19]. Additionally, workload and time pressure were the main causes of emotional and interpersonal stresses among physicians in an ICU [20]. ICU physicians do not encounter many physically demanding activities, but they need to make a lot of LSTs decisions under considerable time pressure, which require ICU physicians’ mental demands. Studies also showed that physicians who experienced overload from work had a high risk of psychological syndromes arising in response to the stressors on the job [21], and psychological syndromes were associated with negative attitudes and behaviors towards individual’s work [22].

Although previous studies have found a variety of physician-related factors related to decision-making on a DNR order, studies examining the effect of physicians’ workload on signing DNR orders for ICU patients are rarely conducted. Therefore, this study aimed to examine whether physician workload is associated with the decision made by patients to consent to a DNR order. We hypothesized that physician workload has an influence on the physician’s writing of a DNR order.

## Methods

### Setting

This observational cohort study was performed in the surgical ICUs in a tertiary medical center with more than 2000 beds located at Northern Taiwan. The surgical ICUs were comprised of cardiovascular units (19 beds), a unit of thoracic surgery and neurosurgery (10 beds), general surgery units (27 beds), and a trauma unit (8 beds). The medical services for caring for the surgical ICU patients were shared by a team of physicians comprised of one board-certified surgical intensivist, and one or two house officer. The board-certified surgical intensivist was responsible for all medical care decisions, including discussing the appropriateness of DNR with patients and/or family members, writing a DNR order for the patient, and so on.

### Study design

The medical records of the patients who met the following criteria were retrospectively reviewed: patients who were at the age of 20 or older; admitted to the surgical ICUs with a Therapeutic intervention scoring system (TISS) score; cared for by only one attending physician during their ICU stay; and admitted between June 2011 and December 2013. We collected patient-related variables including age, gender, religious background, education, marital status, working status, residence, the TISS score upon ICU admission, ICU admission diagnosis, the status of writing a DNR order, and the time duration from ICU admission to writing a DNR order. The attending physician-related variables such as age, gender and seniority were collected.

The TISS scoring system developed by Cullen et al. in 1974 [23] has become a widely accepted method for measuring the severity of clinical illness in ICUs [24, 25]. The score ranges from 0 to 174. Higher scores of TISS indicate more severe clinical illness and demand a higher number of therapeutic interventions and treatments. Based on the 50 APACHE II (Acute Physiology and Chronic Health Evaluation II) diagnostic categories [26], we collapsed the surgical ICU admission diagnosis into only 4 categories: (1) non-operative, cardiac failure/insufficiency; (2) non-operative, others; (3) post-operative, major surgery; and (4) post-operative, others.

We estimated attending physician workload which was defined as the average number of patients each attending physician cared for per day. It was calculated as the sum of the patient-days of patients each attending physician cared for divided by the total number of days in data collection period. Based on the average number of patients they cared for per day, the attending physicians were divided into two workload groups: (1) those who cared for more than or equal to one patient per day; and (2) those who cared for less than one patient per day. The outcome variable in this study was the status of writing a DNR order. Furthermore, the incidence rate of writing a DNR order for each attending physician was calculated as the number of DNR decision divided by the sum of the patient-days for all patients each attending physician cared for.

### Statistical analysis

**Fig. 1:**
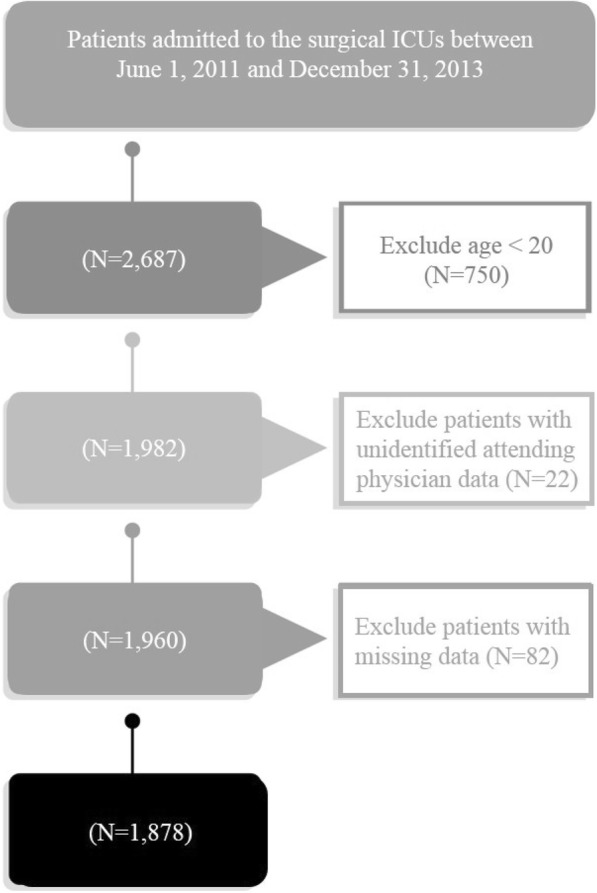
Participant selection

Data analysis was conducted using the data of patients who had no missing data in all variables (Fig. 1). We used descriptive statistics to analyze the characteristics of patients and physicians. All data were expressed as the frequency (percentage) or mean ± standard deviation. Continuous variables between the two groups of physicians were compared using Student’s t-test. Categorical variables between the two groups were compared using Chi-square test. We used Pearson’s correlation coefficient for examining the linear relationship between the incidence rate of writing a DNR order for each attending physician and the average number of patients each attending physician cared for per day.

Cox proportional hazards regression analysis was conducted to examine the influence of the attending physician’s workload on his/her writing a DNR order by adjusting for the combined effect of multiple factors. Harrell’s C-statistic was used to assess the discriminatory ability of the Cox proportional hazards regression model [27]. Kaplan–Meier survival curves of the two different attending physician workload groups were developed to compare the time from surgical ICU admission to writing a DNR order for patients. A DNR order written was considered as an “event” and the ICU discharge was considered as “censored” in the survival analyses. Differences between the two Kaplan-Meier curves were tested using log rank tests.

A *p* value of less than or equal to 0.05 was considered statistically significant. All statistical analyses were conducted with SAS 9.4 (SAS Institute Inc., Cary, NC, USA). This study was approved by the Research Ethics Committee (REC) in National Taiwan University Hospital (20140308RINC).

## Results

A total of 1878 patients were enrolled. The majority of the participants in this study were males (67.41%) and the average age was 61.72 years (standard deviation = 15.11). Most of them were married (76.73%) and had an education level of high school or below (60.17%). Moreover, 37.91% of the participants were working fulltime, and 5.22% were from rural areas. The mean TISS score of the 1878 participants was 32.01 (standard deviation = 10.80). Approximately 48% of patients had the admission diagnosis of “non-operative, cardiac failure/insufficiency”. The average length of stay in surgical ICU was 6.43 days (standard deviation = 13.23). Among the 1878 patients, 120 (6.4%) had a DNR order written during their surgical ICU stay. The average length of time from ICU admission to writing a DNR order was 20.31 days (standard deviation = 19.31).

**Fig. 2:**
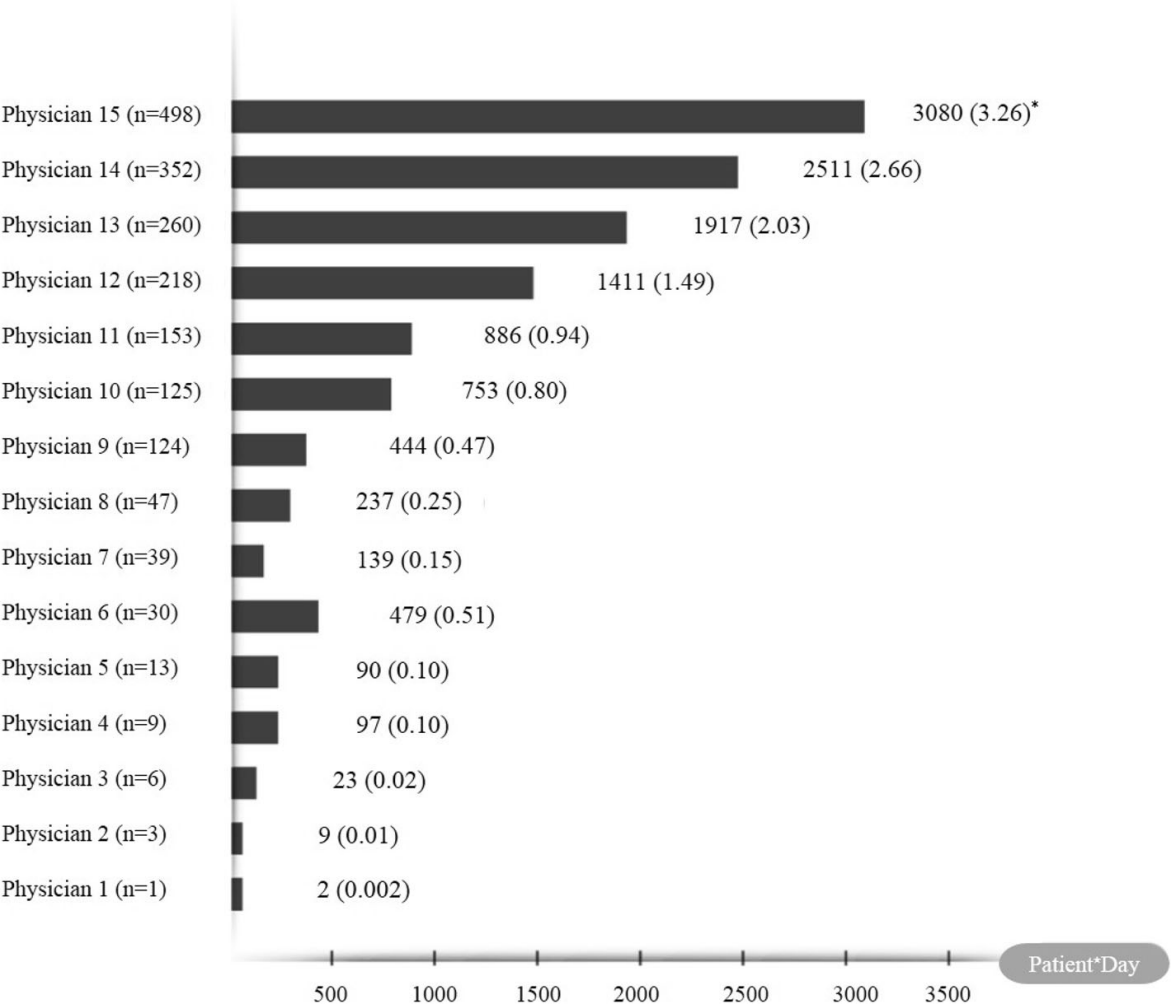
Patient-Day for each attending physician

**Fig. 3:**
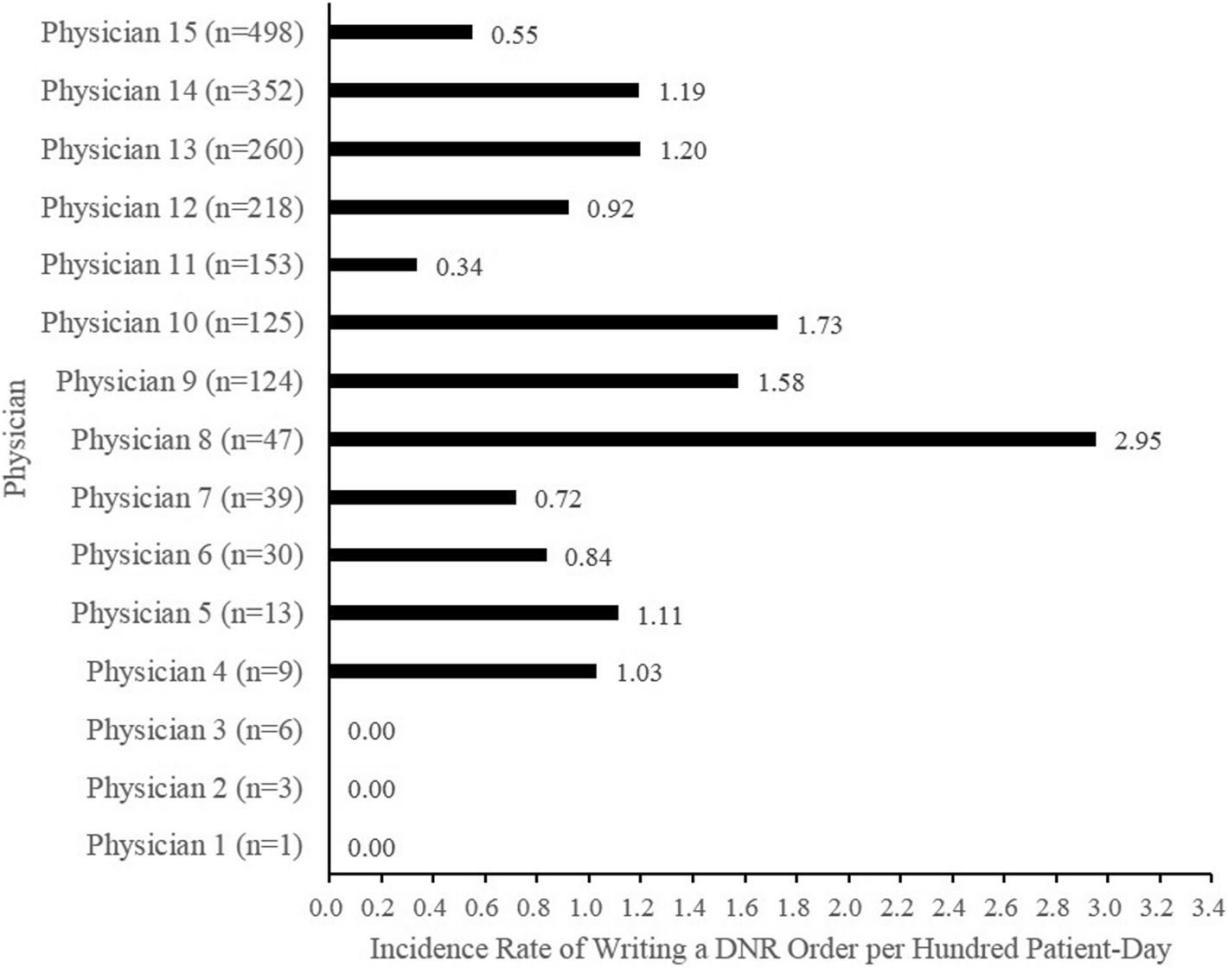
Incidence rate for writing a DNR order for each attending physician

**Fig. 4:**
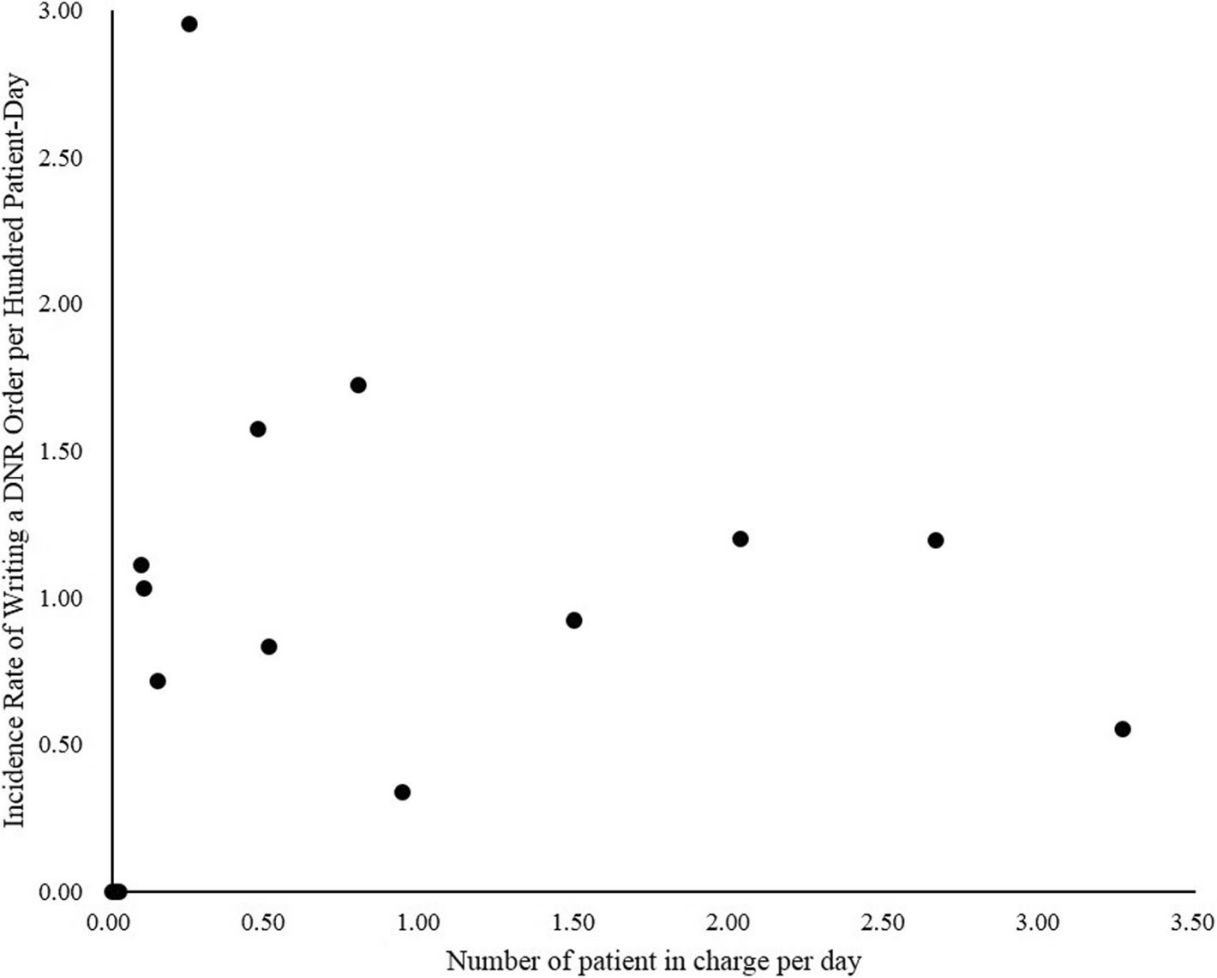
Scatterplot for the relationship between the number of patients in charge and incidence rate of writing a DNR order for each attending physician

All 15 attending physicians who cared for 1878 patients were male, and their average age upon the beginning of data collection period was 50.25 years (standard deviation = 7.51). The total number of patients each physician cared for during data collection period ranged from 1 to 498. Figure 2 shows the total number of patient-days for each attending physician during data collection period, and the average number of patients each attending physician cared for per day during data collection period. Figure 3 shows the incidence rate of writing a DNR order for each attending physician, ranging from 0 to 2.95%. Furthermore, the scatter plot for the relationship between the average number of patients each attending physician cared for per day and the incidence rate of writing a DNR order for each attending physician was shown in Fig. 4, and the Pearson’s correlation coefficient between them was 0.06 (*p* = 0.85).

When compared to the patients cared for by the attending physician with less workload, the patients cared for by the attending physician with heavier workload were married (*p* < 0.01), more likely to have an education level of college or above (*p* = 0.01), admitted to the surgical ICU with a higher TISS score (*p* < 0.01), and more likely to have the admission diagnosis of “non-operative, cardiac failure/insufficiency” (*p* < 0.01) (Table 1).

**Table 1:** Characteristics of patients in different physician workload groups

	Physician workload (the number of patients physician cared for per day)		*P* value
	≧1 (*n* = 1328)	< 1 (*n* = 550)	
	N (%)	N (%)	
Gender			0.98
Male	895 (67.40)	371 (67.46)	
Female	433 (32.60)	179 (32.54)	
Age, years (Mean ± SD)	61.91 ± 14.50	61.26 ± 16.48	0.42
Religion			0.19
Others	633 (47.67)	286 (52.00)	
Buddhist/Daoist	614 (46.23)	237 (43.09)	
Christian/Catholic	81 (6.10)	27 (4.91)	
Education, years			0.01
> 12	425 (32.00)	142 (25.82)	
1–12	771 (58.06)	359 (65.27)	
0	132 (9.94)	49 (8.91)	
Marital status			< 0.01
Married	1053 (79.29)	388 (70.54)	
Others	275 (20.71)	162 (29.46)	
Working fulltime			0.87
Yes	505 (38.03)	207 (37.64)	
No	823 (61.97)	343 (62.36)	
Residence			0.33
Rural area	65 (4.90)	33 (6.00)	
Urban area	1263 (95.10)	517 (94.00)	
Diagnosis			< 0.01
Non-operative, cardiac failure/insufficiency	672 (50.60)	230 (41.82)	
Non-operative, others	58 (4.37)	52 (9.45)	
Post-operative, major surgery	412 (31.02)	131 (23.82)	
Post-operative others	186 (14.01)	137 (24.91)	
TISS (Mean ± SD)	32.74 ± 10.13	30.25 ± 12.10	< 0.01
Length of surgical ICU stay, days (Mean ± SD)	6.72 ± 11.13	5.74 ± 17.27	0.15

Abbreviations: *DNR* do-not-resuscitate, *TISS* Therapeutic Intervention Scoring System, *ICU* Intensive care unit, *SD* Standard deviation

The results of the multivariate Cox proportional hazards model are shown in Table 2. All patient-related variables were put into the model for adjustment. Regarding to the attending physician-related variables, since all attending physicians were male, and their age was highly correlated with their seniority, only physician’s age and workload were included in the model. After adjusting for the potential confounding variables, the hazard of writing a DNR order for their patients cared for by the attending physicians with a heavier workload significantly decreased by 41% as compared to the hazard of writing a DNR order for their patients cared for by the attending physicians with less workload (hazard ratio = 0.59, *p* = 0.01). In addition, the factors associated with writing a DNR order as determined by the Cox model were non-operative, cardiac failure/insufficiency diagnosis (hazard ratio = 1.71, *p* = 0.05) and the TISS score (hazard ratio = 1.02, *p* = 0.03). Harrell’s C-statistic for the model was 0.73, indicating an acceptable discrimination.

**Table 2:** The multivariate Cox proportional hazards model of writing a DNR order

	Hazard Ratio^*^	95% CI for Hazard Ratio		*p* value
		Lower	Upper	
Gender				
Male	0.93	0.60	1.44	0.75
Female	1.00	–	–	–
Age, years	1.01	0.99	1.03	0.14
Religion				
Others	0.62	0.29	1.32	0.21
Buddhist/Daoist	0.63	0.29	1.35	0.23
Christian/Catholic	1.00	–	–	–
Education, years				
> 12	0.79	0.37	1.67	0.54
1–12	0.71	0.37	1.34	0.29
0	1.00	–	–	–
Marital status				
Married	0.84	0.55	1.28	0.41
Others	1.00	–	–	–
Working fulltime				
Yes	0.77	0.47	1.25	0.29
No	1.00	–	–	–
Residence				
Rural area	1.96	0.93	4.14	0.08
Urban area	1.00	–	–	–
Diagnosis				
Non-operative, cardiac failure/insufficiency	1.71	1.00	2.91	0.05
Non-operative, others	2.30	0.87	6.06	0.09
Post-operative, major surgery	0.82	0.40	1.65	0.57
Post-operative others	1.00	–	–	–
TISS	1.02	1.00	1.03	0.03
Physician workload (number of patients physician cared per day)				
≧1	0.59	0.39	0.89	0.01
< 1	1.00	–	–	–
Physician age, years	0.99	0.97	1.02	0.54

Abbreviations: *TISS* Therapeutic Intervention Scoring System, *CI* confidence interval

^*^Adjusted for all above variables

**Fig. 5:**
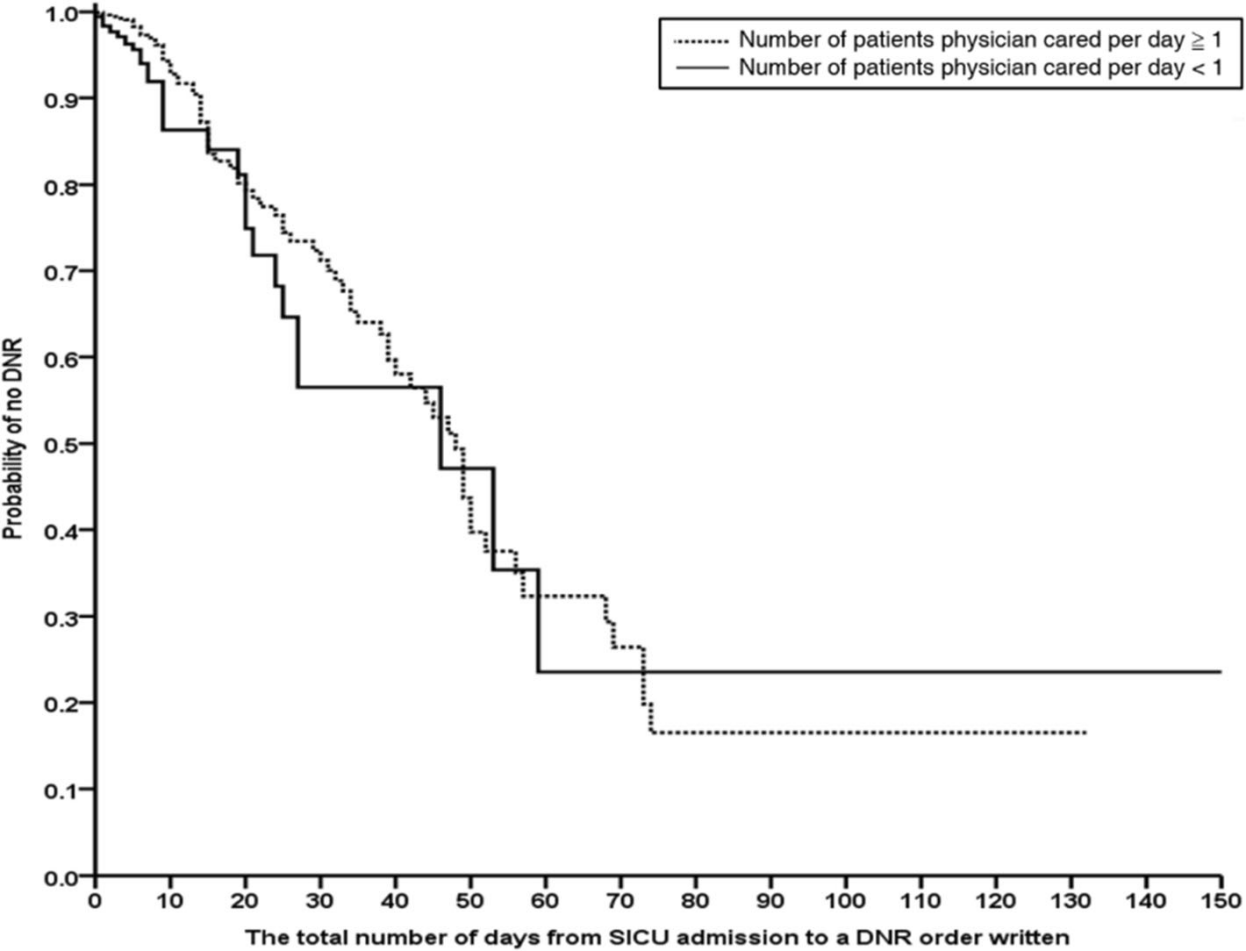
Probability of writing a DNR order

We summarized the significant difference between the two groups of attending physicians’ workloads using Kaplan-Meier analysis in Fig. 5. Attending physicians who cared for greater than or equal to one patient per day during the data collection period were less likely to write a DNR order for their patients than those who cared for less than one patient per day (log-rank chi-square = 5.72, *p* = 0.02).

## Discussion

### Main outcomes

This study examined the influence of attending physician workload on signing a DNR order in ICUs. We found that, after adjusting for other confounding variables, the attending physician with a heavier workload as indicated by the average number of patients he/she cared for per day were less likely to write a DNR order for his/her patients.

### Generalizability

In this study, we retrospectively reviewed 1878 patients’ medical records. Among them, 6.4% of the patients had a DNR order written during their surgical ICU stay. The result was similar to those reported in the literature. Zimmerman et al. reported that the rate of DNR orders ranged from 0.4 to 13.5% in ICU admissions at 13 hospitals [28]. According to a multicenter study conducted in 42 medical centers in the United States, 9% of the 17,440 ICU patients had DNR orders written [29]. Nathan et al., based on the data derived from the National Study on the Costs and Outcomes of Trauma, found that across the ICUs of 68 medical centers, 7% of the 6765 patients had a DNR order [8]. Hence, the percentage of patients with DNR orders in our study was similar to several prior studies.

In addition, the result of this study showed that an admission diagnosis of “non-operative, cardiac failure/insufficiency” and the severity of clinical illness as indicated by the TISS scores were positively associated with writing a DNR order. Our findings were consistent with several prior studies [8, 30, 31]. Phillips et al., using the data derived from the SUPPORT (Study to Understand Prognoses and Preferences for Outcomes and Risks of Treatments) project, found that the diagnosis and severity of clinical illness upon ICU admissions were associated with a DNR order in patients hospitalized with serious illnesses [31].

As indicated by the percentage of writing a DNR order, the ICU admission diagnosis, and the severity of clinical illness, the generalizability of the results of this study may be as good as several prior studies in the literature.

### Physician’s age and do-not-resuscitate orders

In this study, the age of the physician was not significantly associated with writing DNR orders for patients. This finding was in line with the result reported by Giannini et al. that physician age did not have significant influence on the end-of-life treatment decisions [32]. In contrast, some studies found that physician age was a significant factor associated with treatment decisions. Alemayehu et al. found that older physicians were more likely to choose less vigorous treatments [33]. On the other hand, Christakis and Asch found that younger physicians were more strongly in favor of withdrawing LSTs [34]. In spite of the fact that physicians can influence LST decision-making, the influence of physician age on LST decision-making remains controversial.

### Physicians’ workload and do-not-resuscitate orders

We found that the physicians who were stressed with heavier workloads as indicated by caring for more patients were significantly associated with a lower likelihood of writing a DNR order for their patients. Several possibilities may account for this result:

Firstly, the physicians’ lack of time to carefully deliberate the appropriateness of writing a DNR order for the patient may relate to this finding. Since most DNR discussions are prompted by physicians, whether they bring the DNR discussion up can significantly influence patients’/surrogates’ DNR decisions [35, 36]. Physicians who were tasked with caring for a higher volume of patients per day might have had less time to carefully deliberate whether or not cardiopulmonary resuscitation is useful or harmful. As a result, the physicians were less likely to bring the DNR discussion up, and therefore the patients/surrogates were less likely to consent to a DNR order.

Secondly, the physicians’ burnout-associated negative attitudes towards patients may account for this finding. Heavy workload was the main cause of high levels of burnout symptoms among ICU physicians [20, 37]. Since burnout is described as a prolonged response to chronic emotional and interpersonal stress on the job [38], the physicians who were unable to cope with the stress from heavier workloads were more likely to be emotionally and interpersonally exhausted, and developed negative attitudes toward their patients [39]. The physicians, therefore, would be reluctant to visit patients/surrogates and initiate the DNR discussion.

Thirdly, physicians who were tasked with a heavier workload were associated with poor quality of communication with patients/surrogates [40], and were also negatively associated with empathy [41]. For a physician with heavier workload providing intensive and critical care to his/her patients, communicating with the patients/surrogates about LSTs and decision-making such as consenting to a DNR order may not be a compelling task and of low priority. The physicians lacking empathy and a good quality of communication with patients/surrogates were less likely to attempt to initiate the DNR discussion with patients/surrogates [42], or to bring up a successful DNR discussion to achieve the goal of medical care [43]. Therefore, patients/surrogates would be less likely to consent to a DNR order.

Specific education training on EOLC which focuses on issues of DNR orders is needed to give physicians, especially those who are caring for more patients, the confidence and skill to communicate positively with patients/surrogates. Moreover, it is not only front line healthcare workers who need additional training but also healthcare workers not on the frontline. In ICU, since attending physicians serving on the frontline may be exposed to a high level of stress with heavier workloads and challenges [44], a DNR discussion with patients/surrogates is encouraged to be initiated by well-trained healthcare workers serving non-frontline positions [45].

Since excessive workloads may prevent physicians from having sufficient time to assess patients, and may decrease quality of patient care [46], stress management training programs in reducing work stress and risk of psychological syndromes due to high workload are highly suggested for physicians. Person-directed (e.g. cognitive behavioral therapy, relaxation, music making, massage, and so on) and work-directed (e.g. attitude change and communication, support from colleagues, participatory problem solving and decision-making, changes in work organization, and so on) intervention strategies for preventing work stress in healthcare workers have been proposed in the previous studies [47]. Hence, for those physicians who suffered high workloads, it is useful to apply suitable intervention strategies as early as possible for promoting physicians’ psychological health as well as encouraging the DNR discussion with patients.

### Strengths and limitations

We reported that attending physician workload indicated by the average number of patients he/she cared for per day was significantly associated with writing a DNR order, which was never examined in the past. However, there are some limitations in this study:

Firstly, our findings may not reflect the situation existing in other medical institutions. In addition, this study was conducted in surgical ICUs. It may be possible to have different findings if examining the same issue in medical ICUs. In addition, since attitudes and perspectives about DNR may vary in different places and healthcare institutions [48], this study’s results are closely related to the most current and comprehensive interpretation of DNR in Taiwan.

Secondly, some insufficiencies in methods might hurt the outcomes of this study. For example, some potential confounding variables, e.g. unmeasured physicians’ variables, were not included in the multivariate Cox proportional hazards regression model. In addition, although the multivariate Cox proportional hazards regression model is most popular for survival data analysis, there may be concerns that our results still suffer from ecological bias.

Thirdly, the DNR order was not always consented to by the patient. Decisions to consent to DNR orders in Taiwan are usually made by family members [49]. This study was limited by the omissions of information that some DNR orders may have been consented to by surrogate decision-makers. However, the omissions did not obscure the value of our study results because this was not usually considered in prior studies on DNR.

Fourthly, the average number of patients an attending physician cared for per day was used as an indicator of workload in this study. However, the average number of patients an attending physician cared for per day may not comprehensively represent the workload. For example, some physicians caring for a fewer number of patients may need to do administrative or research tasks as well. Nonetheless, due to a lack of a well-developed and widely-recognized way to quantify the administrative and research workload, the average number of patients a physician cared for per day and each physician’s total number of patient-days may still have academic merit to provide sufficient information on workload. In addition, patients usually require the most work when they arrive at ICUs and when they leave ICUs. Simply seeing the workload by taking the average number of patients cared for a day may be of concerns.

## Conclusion

Our study reported that attending physicians who suffered from heavier workloads as indicated by caring for more patients per day were less likely to write a DNR order. Our findings highlight the need to take multidisciplinary actions for attending physicians suffering from heavy workloads. Changes in the work environmental factors along with stress management programs to improve physicians’ psychological well-being as well as the quality of care provided to patients are warranted. Future studies should focus on examining the association between physician workload and the likelihood of writing a DNR order for patients through qualitative and quantitative research. Even if physicians are stressed with heavy workloads, educational interventions are still important and should be executed to facilitate the discussion between medical professionals/other healthcare team members and patients/family members about goals of care and preferences regarding resuscitation.

## Abbreviations

APACHE II: Acute Physiology and Chronic Health Evaluation II
DNR: Do-not-resuscitate
EOLC: End-of-life care
ICU: Intensive care units
LST: life-Supporting treatment
PSDA: Patient Self-Determination Act
REC: Research Ethics Committee
SUPPORT: Study to Understand Prognoses and Preferences for Outcomes and Risks of Treatments
TISS: Therapeutic Intervention Scoring System
